# Comorbidity of depression and diabetes: an application of biopsychosocial model

**DOI:** 10.1186/s13033-016-0106-2

**Published:** 2016-12-03

**Authors:** Tesfa Dejenie Habtewold, Md. Atiqul Islam, Yosef Tsige Radie, Balewgizie Sileshi Tegegne

**Affiliations:** 1 Department of Nursing, Debre Berhan University, 445, Debre Berhan, Ethiopia; 2 Department of Epidemiology and Rob Giel Research Center, University of Groningen, University Medical Center Groningen, Groningen, The Netherlands; 3 Department of Statistics, Shahjalal University of Science and Technology, Sylhet, 3114 Bangladesh; 4 Centralized School of Nursing and Midwifery, Addis Ababa University, Addis Ababa, Ethiopia; 5 Department of Epidemiology, University of Groningen, University Medical Center Groningen, Groningen, The Netherlands; 6 Department of Public Health, Haramaya University, Dire Dawa, Ethiopia

**Keywords:** Biopsychosocial model, Comorbidity, Depression, Diabetes mellitus

## Abstract

**Background:**

Type 2 diabetes (T2D) is one of the most psychologically demanding chronic medical illness in adult. Comorbidity between diabetes and depression is quite common, but most studies were based on developed country sample. Limited data exists to document biopsychosocial predictors of depressive symptoms in Ethiopian patients. Therefore, the aim of the study was to describe the association of depressive symptoms and T2D and explore the potential underlying associated biopsychosocial risk factors.

**Methods:**

Institution based cross-sectional study was conducted on 276 patient with T2D at diabetic clinic, Black Lion General Specialized Hospital in Ethiopia. Patients were selected using systematic random sampling technique. Depressive symptoms score, which constructed from a validated nine-item Patient Health Questionnaire (PHQ-9), was an outcome variable. Finally, significant associated factors were identified using multiple linear regression analysis with backward elimination procedure. Statistical Package for Social Science (SPSS) version 22.0 (IBM SPSS Corp.) was used to perform all analysis.

**Results:**

Total of 264 patient data was analyzed with 95.7% response rate. Patients mean (SD) current age and age at diagnosis was 55.9 (10.9) and 43.9 (10.9) years, respectively. Patients waist circumference (mean ± SD) was 98.9 ± 11.1 cm. The average PHQ-9 score was 4.9 (SD 4.1) and fasting blood glucose was 166.4 (SD 73.2). Marital status (divorced), occupation (housewife), diabetic complication (nephropathy), negative life event in the last six months, and poor social support significantly associated with increased mean PHQ-9 score after adjustment for covariates. Whereas not fearing diabetic-related complication and death significantly lower mean PHQ-9 score.

**Conclusion:**

Biopsychosocial variables including marital status, negative life event in the last 6 months, occupation, diabetic complication, and poor social support significantly increase average depressive symptoms score. Evidence-based intervention focusing on these identified biopsychosocial factors are necessary to prevent the development of depressive symptoms.

**Electronic supplementary material:**

The online version of this article (doi:10.1186/s13033-016-0106-2) contains supplementary material, which is available to authorized users.

## Background

Diabetes mellitus (DM) has been affecting millions of people from all over the world. In 2013, 382 million people had diabetes; this estimate is expected to rise to 592 million by 2035 [1, 2]. More than 77% of morbidity and 88% of mortality due to DM occur in low and middle-income countries. In Ethiopia, the prevalence of diabetes was 0.34–5.0% [3, 4]. During the last decades, the comorbidity of mental disorders with chronic health conditions have emerged as a topic of considerable clinical and policy interest. Due to complex nature of disease pathophysiology, cause, and treatment, type 2 diabetes (T2D) is considered one of the most psychologically demanding chronic medical illness in an adult patient [5, 6]. In spite of this, up to 45% of cases of comorbid mental disorder and severe psychological distress were poorly identified and inadequately treated among patients with diabetes in sub-Saharan Africa [7, 8]. The prevalence of psychiatric disorders in diabetic patients may reach 84% for mood disorders and 80% for anxiety disorders [9, 10]. Based on a study report by Ana Claudia and colleagues the most prevalent comorbid disorders were generalized anxiety disorder (21%), dysthymia (15%), social phobia (7%), lifelong depression (3.5%), panic disorder (2.5%), and risk of suicide (2%) [10]. Depression was among the most common neuropsychiatric disorders in patients with T2D [8].

Thomas Willis, British physician, recognized the association between depression and diabetes since 17th century [11]. Epidemiologically, one in every four patient with T2D develops clinically significant depression [12]. The estimated lifetime prevalence of depression was higher in women (21%) [13]. The prevalence of depression in T2D patient was 5.5–49.6% [10, 14–22]. Even though most studies was on Western samples, there have been emerging studies in developing countries including Ethiopia [16, 23–25]. A cross-sectional study by Erkie et al. described depression was diagnosed in 64.8% of T2D outpatient [23]. The exact mechanisms of relationship are elusive, and models for the associated factors are multidimensional.

**Fig. 1:**
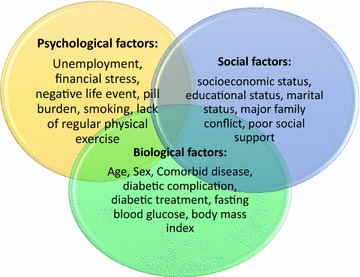
Engle’s biopsychosocial model of health and disease adapted for our study

Engel’s [26] biopsychosocial model of health and illness is a model for clinical practice and research for psychologists, nurses, physicians, and social workers [27]. American Psychiatric Association and American Board of Psychiatry and Neurology have officially approved Engel’s model [28, 29]. According to Engel’s model any disease such as depression [30–33] caused by biological (physiological or genetic predispositions), psychological (health beliefs and lifestyle) and social factors (family relationships, socioeconomic status, and social support). The model reveals the interaction of this factor to create patient’s state of mind and body [34, 35] (Fig. 1).

T2D patients were poorly diagnosed and inadequately treated in sub-Saharan Africa [8]. In general, the data is limited, and the conclusion seems inadequate to identify biopsychosocial risk factors of depressive symptoms in Ethiopian diabetic patients. In the present study, we aimed to describe the association of depressive symptoms and T2D, and explore the potential underlying associated risk factors.

## Methods

### Study design and population

We conducted an institution based cross-sectional study among T2D outpatients on regular follow-up at Black Lion General Specialized Hospital from February to April 2013.

### Determinants and covariables

The outcome variable was depressive symptoms score. The explanatory variables were biological factors: age, sex, comorbid disease, diabetic complication, diabetic treatment, fasting blood glucose, body mass index; psychological factors: unemployment, financial stress, negative life event, polypharmacy, smoking, lack of regular physical activity, perceived fear of complication and death, perceived high healthcare cost; and social factors: socioeconomic status, educational status, marital status, major family conflict, poor social support. We defined polypharmacy as taking greater than or equal to four prescribed medications per day. Poor social support was defined as lack of support or care from the family, friends, and neighbors. Another studied factor was, which referred to an event such as accident and death in the last six months that leads to a feeling of stress or anxiety, negative life event. Perceived fear of complication and death was defined as individual feeling or opinion about his/her illness and related complication. Perceived high healthcare cost was defined as personal feeling or idea about the expense of diabetes treatment. Physical activity was defined as doing any aerobic exercise 3–5 times per week at least for 30 min.

### Sampling and data collection

Patients were chosen based on three criteria: T2D diagnosis at least for one year, age ≥20 years old, capable of independent communication, and signed written informed consent. Patients treated for depression, or other psychological illnesses (e.g. anxiety or personality disorders) were excluded. Systematic random sampling technique was used to reach individual patients. The data was collected by two trained Nurses from every three patients (sampling interval/k/ = 3). All biological (physiological) data was collected from patients’ medical chart. Face-to-face interview was conducted in the treating private clinics to collect psychological and social data. Twelve patients refused to take part because of lack of interest to participate and a shortage of time. In total 264 cases were included in the final analysis.

### Measuring depressive symptoms

Depression, which refers to symptoms experienced during the last two weeks, was measured by Patient Health Questionnaire (PHQ-9) [36, 37]. The PHQ-9 includes nine items with individual score ranges from 0 (not at all) to 3 (nearly every day). The total sum score ranging from 0 to 27. PHQ-9 scores with cut-off point 5, 10, 15 and 20 represent mild, moderate, moderately severe, and severe depression, respectively [36]. In our study, T2D patients’ depression status was measured by administering a validated Ethiopian version PHQ-9 questionnaire. Gelaye and colleagues showed PHQ-9 internal reliability of 0.81, test re-test reliability of 0.92, sensitivity of 86%, and specificity of 67% [38].

### Statistical analysis

First of all, four cases were not included in our analysis because of outlying PHQ-9 score (≥20). Descriptive statistics including mean, standard deviation, percentage, and cross-tabulation was performed for all patient parameters. Univariate linear regression analysis was performed per each biopsychosocial variable. The full model of multiple linear regression included all significant variables. Finally, significantly associated factors were identified by backward elimination procedure. QQ-plot, histogram and scatter plot of ‘Standardized residuals’ against ‘Standardized predicted values’ were used to check the assumptions of linearity of relationships, normal distribution and homoscedasticity of residuals for the final model. Two-tailed test at 5% level of significance was used for all association test. Statistical Package for Social Science (SPSS) version 22.0 (IBM SPSS Corp.) was used to perform all analysis. The study was adherent to the STROBE criteria.

## Results

### Biopsychosocial characteristics of patients

Total of 264 patient data was analyzed with 95.7% response rate. Patients mean (SD) current age and age at diagnosis was 55.9 (10.9) and 43.9 (10.9) years, respectively. Also, patients waist circumference (mean ± SD) was 98.9 ± 11.1 cm while patients family median monthly income was 750 Ethiopian Birr (651–1400). The average PHQ-9 score was 4.9 (SD 4.1) and fasting blood glucose was 166.4 (SD 73.2). The mean ± SD of PHQ-9 score was 6 ± 4.7 in female, 7.3 ± 5.7 in divorced, 6.6 ± 4.5 in educational level of grade 1–8, and 6.77 ± 5.3 in housewife patient. The mean ± SD of number of comorbid diseases and body mass index was 1.1 ± 0.9 and 25.4 ± 3.7, respectively (Table 1).

**Table 1:** Distribution of patients PHQ-9 score and fasting blood glucose by biopsychosocial characteristics of patients

Variable	Categories	n (%)	PHQ-9 score mean (± SD)	Fasting blood sugar mean (±SD)
Gender, female		140 (53.0)	6.0 (4.77)	181.30 (79.94)
Residence, Addis Ababa		228 (86.4)	5.07 (4.35)	166.13 (71.78)
Marital status	Married	183 (69.3)	4.68 (4.17)	164.4 (70.67)
	Divorced	24 (9.1)	7.3 (5.75)	188.94 (94.31)
	Widowed	48 (18.2)	5.92 (4.73)	162.04 (66.48)
Religion	Orthodox christian	213 (80.7)	5.48 (4.74)	167.64 (72.76)
	Muslim	24 (9.1)	3.71 (3.14)	170.04 (92.81)
	Other religion	5 (1.9)	4.67 (4.04)	159.63 (61.18)
Ethnicity	Amhara	151 (57.2)	5.23 (4.71)	168.71 (74.05)
	Oromo	40 (15.2)	5.8 (4.54)	165.65 (77.42)
	Others	19 (7.2)	4.93 (4.33)	164.36 (70.94)
Educational status	No formal education	51 (19.4)	6.1 (5.17)	179.57 (81.79)
	Grade 1–8	16 (6.1)	6.63 (4.53)	171.11 (78.94)
	Grade 9–12	59 (22.3)	4.42 (4.08)	165.66 (63.11)
	College/university	89 (33.7)	4.26 (4.25)	157.81 (70.59)
Occupation	Civil servant	47 (17.8)	4.74 (4.71)	172.51 (59.36)
	House wife	47 (17.8)	6.77 (5.26)	183.45 (71.56)
	Private worker	38 (14.4)	4.29 (3.84)	162.95 (93.59)
	Pensioned	58 (22.0)	4.69 (4.48)	142.0 (56.18)
	No employment	48 (18.2)	5.85 (4.14)	172.9 (80.15)
	Others	16 (6.1)	4.81 (4.53)	178.54 (79.98)
Diabetes treatment	Single insulin injection	108 (40.9)	5.78 (4.99)	162.44 (83.22)
	Combined insulin injection	12 (4.5)	5.92 (5.14)	218.17 (104.57)
	Insulin plus oral hypoglycemic	30 (11.4)	5.23 (3.61)	167.67 (58.89)
	Oral hypoglycemic	114 (43.2)	4.65 (4.29)	165.85 (61.25)
Comorbid disease (N = 180)	Cardiovascular disease	141 (78.3)	5.57 (4.68)	162.81 (68.43)
	Respiratory disease	17 (9.4)	5.41 (4.43)	151.53 (66.82)
	Renal disease	13 (7.2)	5.54 (4.31)	158.46 (58.13)
	Neurologic disease	4 (2.2)	6.75 (6.13)	131.5 (35.48)
	Others comorbidity	80 (44.4)	6.04 (5.09)	174.59 (72.01)
Diabetic complication (N = 201)	Diabetic retinopathy	140 (69.7)	5.79 (4.67)	166.76 (70.51)
	Diabetic nephropathy	69 (34.3)	7.09 (5.16)	176.43 (72.36)
	Diabetic neuropathy	83 (41.3)	6.14 (4.59)	172.41 (81.17)
	Sexual dysfunction	69 (34.3)	4.70 (4.77)	162.29 (69.88)
Physical disability		132 (50.0)	5.80 (4.68)	169.61 (75.71)
Poor social support		95 (37.4)	7.44 (5.03)	167.47 (82.95)
Negative life event		88 (34.6)	6.64 (4.93)	168.75 (84.38)
Physical activity		55 (22%)	3.80 (3.53)	165.98 (82.69)
Perceived fear of complication and death		178 (70.1)	5.78 (4.74)	167.24 (71.88)
Perceived high health care cost of diabetes		192 (75.6)	5.35 (4.75)	168.15 (75.95)

*SD* standard deviation

### Univariate linear regression test of association

Patient mean PHQ-9 score was significantly increased by 1.4 (95% CI 0.4–2.4) in female, 2.2 (95% CI 0.7–3.7) in divorced, and 1.7 (95% CI 0.4–3.0) in housewife. One unit increase in number of comorbidities was associated with a 0.6 unit (p = 0.04) increase in PHQ-9 score. One unit increase in number of diabetic complication was associated with a 0.5 unit (p = 0.02) increase in PHQ-9 score. Increased age at diagnosis (i.e. late-onset diabetes), increased monthly family income, high educational status (college or university), doing physical activity and not fearing diabetes-related complication and death significantly lower mean PHQ-9 score (Table 2).

**Table 2:** Univariate linear regression test examining the association between biopsychosocial variables and PHQ-9 score of patients

Variables (reference category)	β (95% CI)	p value
Current age	−0.03 (−0.08, 0.02)	0.21
Age at diagnosis	−0.05 (−0.1, −0.001)	0.04
Female gender (male)	1.4 (0.4, 2.4)	0.01
Addis Ababa residence (outside Addis Ababa)	−0.5 (−2.0, 1.0)	0.54
Monthly family income (ETB)	−0.001 (−0.001, −0.0002)	<0.001
Marital status (married)		
Divorced	2.2 (0.7, 3.7)	0.01
Widowed	0.3 (−1.1, 1.6)	0.70
Religion (orthodox christian)		
Muslim	−1.4 (−3.1, 0.3)	0.11
Other religion	−0.4 (−2.0, 1.3)	0.67
Ethnicity (others)		
Amhara	−0.2 (−1.2, 0.8)	0.67
Oromo	1.0 (−0.4, 2.4)	0.17
Educational status (no formal education)		
Primary school (1–8)	1.8 (0.7, 3.0)	0.002
Secondary school (9–12)	−0.7 (−1.9, 0.5)	0.23
College/university	−1.4 (−2.4, −0.3)	0.01
Occupation (others)		
Civil servant	−0.7 (−2.0, 0.6)	0.28
House wife	1.7 (0.4, 3.0)	0.01
Private worker	−0.8 (−2.2, 0.6)	0.26
Pensioned	−0.7 (−2.0, 0.5)	0.23
No employment	1.1 (−0.7, 2.2)	0.10
Waist circumference	0.01 (−0.4, 0.1)	0.74
Duration of diabetes	0.03 (−0.03, 0.1)	0.35
Duration of diabetes treatment	0.03 (−0.04, 0.1)	0.36
Fasting blood glucose	0.01 (−0.001, 0.01)	0.09
Number of co-morbidity	0.6 (0.02, 1.1)	0.04
Number of prescribed medication administration per day	0.2 (−0.05, 0.4)	0.13
Number of diabetic complication	0.5 (0.1, 0.9)	0.02
Body mass index	0.1 (0.03, 0.3)	0.04
Combined insulin injection (single insulin injection)	1.0 (−1.9, 0.4)	0.42
Insulin injection plus oral hypoglycemic (single insulin injection)	0.3 (−1.3, 1.9)	0.72
Oral hypoglycemic agent (single insulin injection)	−0.9 (−2.0, 0.1)	0.08
Cardiovascular disease	0.5 (−0.5, 1.5)	0.33
Respiratory disease	0.5 (−1.6, 2.5)	0.66
Renal disease	0.6 (−1.7, 2.9)	0.62
Neurologic disease	1.8 (−2.3, 5.9)	0.39
Others comorbidity	0.7 (−0.4, 1.8)	0.22
Diabetic retinopathy	1.0 (0.02, 2.0)	0.04
Diabetic nephropathy	2.0 (0.8, 3.1)	0.001
Diabetic neuropathy	1.1 (0.03, 2.2)	0.04
Sexual dysfunction	−1.0 (−2.2, 0.1)	0.08
Physical disability	1.2 (0.2, 2.2)	0.02
Poor social support	3.1 (2.1, 4.1)	<0.001
Doing physical activity	−1.5 (−2.7, −0.3)	0.01
Fear of diabetic complication and death (no perception at all)	1.6 (0.6, 2.7)	0.003
Not fearing diabetic complication and death (no perception at all)	−2.0 (−3.1, −0.9)	<0.001
High health care cost (no perception at all)	0.4 (−0.7, 1.5)	0.50
Not high health care cost (no perception at all)	−0.7 (−2.0, 0.5)	0.26
Negative life event in the last 6 months	2.0 (0.9, 2.9)	<0.001

*β* regression coefficient; *CI* confidence interval

### Multiple linear regression tests of association

All significant biopsychosocial variables, Table 3, in the final model together explained about 25.3% of the variability of patients PHQ-9 score. Divorce, housewife, diabetic nephropathy, negative life event, and poor social support were significant risk factors associated with increased PHQ-9 score after adjustment for covariates. However, not fearing diabetic-related complications and death significantly lower PHQ-9 score. Additional file 1: Table S1 presented all confounding factors.

**Table 3:** Multiple linear regression test examining the relation between different biopsychosocial associated factors and PHQ-9 score of patients with T2D mellitus

Variables	β (95% CI)	p value	t value
Divorce	2.0 (0.6, 3.4)	0.004	2.91
Negative life event in the last 6 months	1.3 (0.3, 2.2)	0.009	2.63
House wife	1.7 (0.5, 2.8)	0.005	2.80
Diabetic nephropathy	1.5 (0.4, 2.5)	0.005	2.80
Poor social support	2.4 (1.5, 3.4)	<0.001	5.07
Not fearing diabetic-related complications and death	−1.5 (−2.5, −0.5)	0.003	−2.97

*β* regression coefficient; *CI* confidence interval

The final model reasonably fulfilled three assumptions: linearity of relationship (Additional file 1: Figure S1), homoscedasticity (Additional file 1: Figure S2), and normal distribution (Additional file 1: Figure S3) assumptions. For further information, residual statistics table (Additional file 1: Table S2) accompanied as well.

## Discussion

This study examined biopsychosocial factors associated with comorbid depression in patients with T2D.

In this study diabetic nephropathy, biologic factor consistent with other studies [39, 40], significantly increased the risk of depression. However, several other studies recognized gender [16, 17, 21, 41–43], age [16, 17, 20, 44, 45], diabetic treatment [21, 46, 47], body mass index [21, 48], fasting plasma glucose [17–19, 49, 50], poor diabetes mellitus control [15], number of comorbidities [21, 48, 51, 52], diabetic complications [16, 17, 53, 54], duration of diabetes [23, 45], age at diabetes diagnosis [55, 56], large waist circumference [39], diabetic retinopathy [40], diabetic neuropathy [39, 40, 57, 58], cardiovascular disease comorbidity [39, 40, 59, 60], sexual dysfunction [40] were physiologic (biologic) risk factors that significantly associated with depression.

In this study occupational status (housewife) and experiencing negative life events, psychological factors, significantly increased risk of depression in line with other earlier studies [39, 57, 61]. Interestingly, our final model uncovered not fearing diabetic related complications and death significantly lower risk of depression. On the other hand, depression was associated with diabetes treatment complexity [62], experienced loss of business or crop failure [16], unemployment [44, 47, 52], lack of regular physical activity [14, 21, 47], smoking [21, 48, 63], financial stress [39, 57, 61], poor quality of life [61, 64], and polypharmacy [39, 65].

Finally, we confirmed marital status (divorce) and poor social support, social factors similar to previous studies [19, 21, 57], significantly increased risk of depression. Contrariwise, urban residence [59], low socioeconomic status [19, 20, 42, 47, 66], lower educational status [23, 47, 49, 52], marital status [15, 17, 19, 21, 24, 44], major family conflicts and unavailability of food or medicines [16] were significant associated factors for depression.

Similar to previous studies [39, 58, 59, 67–70], our final model proved risk of depression was not significantly associated with current age, sex, educational status, residence, ethnicity, socioeconomic status, poor body weight control, insulin treatment users, duration of diabetes, obesity, hypertensive disorder, and diabetic retinopathy. Recent studies [39–42, 47–49, 51, 66] found diabetic neuropathy, doing physical activity, diabetic retinopathy, educational status, perceived fear of diabetes-related death and complication, number of diabetic complication, being female, physical disability, increased body mass index, low monthly family income, age at diagnosis, and increased number of co-morbid disease significantly associated with depression. However, our study lacks to confirm this robust fact.

Most of these inconsistencies might be attributed to inadequacies in study design, implementation (i.e. data analysis and sample selection), interpretation (i.e. categorizing and using different cutoff point to diagnose depression), inadequately powered sample groups, and using different depression diagnostic tool. Using dichotomized PHQ-9 score as an outcome variable clearly causes loss of information, loss of power, bias, incomplete correction for confounding factors, and difficulty for robust replication of associated risk factors [71–73]. Similarly, Olivier Naggara and colleagues argued dichotomization is unnecessary for statistical analysis, and continuous variable should be left alone in statistical model [74]. Researchers have used different cut-off point for dichotomizing PHQ-9 score [22, 59] that compromise replication for an unbiased view of the evidence from a particular study.

This study has important public health implication for health care practice in Black Lion General Specialized Hospital and another health facility, where clinician diagnosis of mental illness (depression) rate is low because of high patient load, lack of screening tool, role confusion, and lack of training. Another important barrier to the care of people with mental and physical health problem in lower and middle-income country is the lack of an integrated model for mental and medical health service [75]. We suggest physician or physiotherapist screen mental health and psychiatrist screen physical health of patient. Finally, clinicians should be aware of various factors and use biopsychosocial model to integrate their patient care.

### Strengths and limitations

The strength of this study includes the use of PHQ-9 score as continuous outcome variable. Variables were defined based on Engel’s biopsychosocial model as well. However, this study has certain limitation. Most parameters estimated were biologic (physiologic) factors. This might underestimate the effect of psychological and social factors. Poor social support, which was identified as a highly significant associated factor, was assessed by a single item. Additionally, this study was conducted in one institution that might limit external validity of the finding. This study examined only the associations between the selected variables and PHQ-9 score. Lastly, the role of inflammation and genetic susceptibility for the emergence of depressive symptoms was not assessed.

## Conclusions

In general biopsychosocial variables including marital status, negative life event in the last six months, occupational status, diabetic complication, and poor social support significantly increased risk of depression. Evidence-based intervention focusing on these identified biopsychosocial factors are necessary to prevent the development of depressive symptoms. Our study finding described the effect of various biopsychosocial factors on patient’s mood because depression-related factors frequently missed in people with diabetes [76]. This will improve evidence-based practice for comprehensive management physical and mental illness [77].

## Additional file

**Additional file 1: Table S1.** List of confounding factors that affect the influence of explanatory variables on and PHQ-9 score of patients with type 2 diabetes mellitus. **Figure S1.** Normal P–P plot of regression standardized residual of the final model. **Figure S2.** Scatter plot of regression standardized residual of the final model. **Figure S3.** Histogram of regression standardized residual of the final model. **Table S2.** Residuals statistics for the final model.
