# Supplementary material for: Comorbidity of depression and diabetes: an application of biopsychosocial model
Source: Int J Ment Health Syst. 2016 Dec 3;10:74. doi: 10.1186/s13033-016-0106-2 (PMC5135819; doi:10.1186/s13033-016-0106-2)
Supplement: Supplementary file 1 — Additional file 1: Table S1. List of confounding factors that affect the influence of explanatory variables on and PHQ-9 score of patients with type 2 diabetes mellitus. Figure S1. Normal P–P plot of regression standardized residual of the final model. Figure S2. Scatter plot of regression standardized residual of the final model. Figure S3. Histogram of regression standardized residual of the final model. Table S2. Residuals statistics for the final model. [file 13033_2016_106_MOESM1_ESM.docx]

**Additional files**

Additional file 1: Table S1. List of confounding factors that affect the influence of explanatory variables on and PHQ-9 score of patients with type 2 diabetes mellitus.

| **Variables** | **B (standardized)** | **t-value** | **p-value** | **Partial Correlation** | **Collinearity Statistics** |
| --- | --- | --- | --- | --- | --- |
| Diabetic neuropathy | 0.057 | 1.017 | .310 | 0.064 | 0.951 |
| Doing physical activity | -0.030 | -0.514 | .608 | -0.032 | 0.889 |
| Diabetic retinopathy | 0.027 | 0.465 | .642 | 0.029 | 0.898 |
| College/University education | -0.042 | -0.725 | .469 | -0.046 | 0.887 |
| Perceived fear of death and complication | 0.036 | 0.347 | .729 | 0.022 | 0.268 |
| Number of diabetic complication | 0.067 | 1.016 | .311 | 0.064 | 0.671 |
| Female | 0.068 | 1.120 | .264 | 0.070 | 0.790 |
| Physical disability | 0.076 | 1.334 | .183 | 0.084 | 0.914 |
| Body mass index | 0.080 | 1.408 | .160 | 0.088 | 0.922 |
| Monthly family income | -0.085 | -1.498 | .135 | -0.094 | 0.908 |
| Age at diagnosis | -0.063 | -1.119 | .264 | -0.070 | 0.941 |
| Number of co-morbid disease | 0.096 | 1.740 | .083 | 0.109 | 0.960 |
| Primary school (1-8) education | 0.104 | 1.868 | .063 | 0.117 | 0.938 |


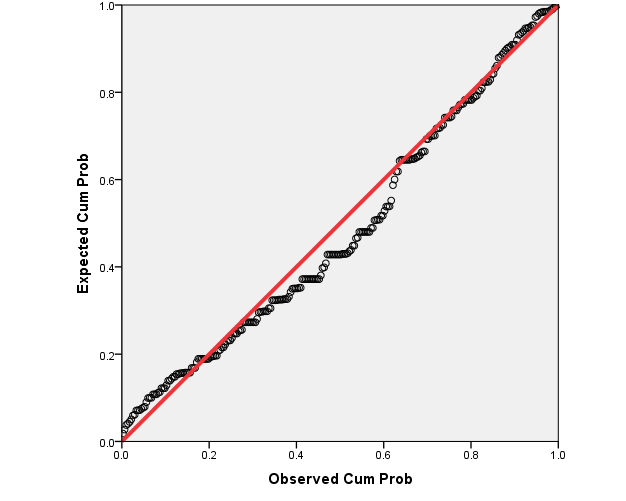


Additional file 1: Figure S1. Normal P-P plot of regression standardized residual of the final model


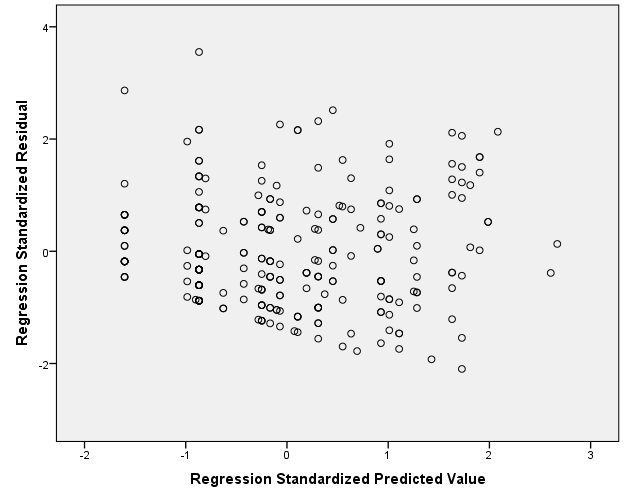


Additional file 1: Figure S2. Scatter plot of regression standardized residual of the final model


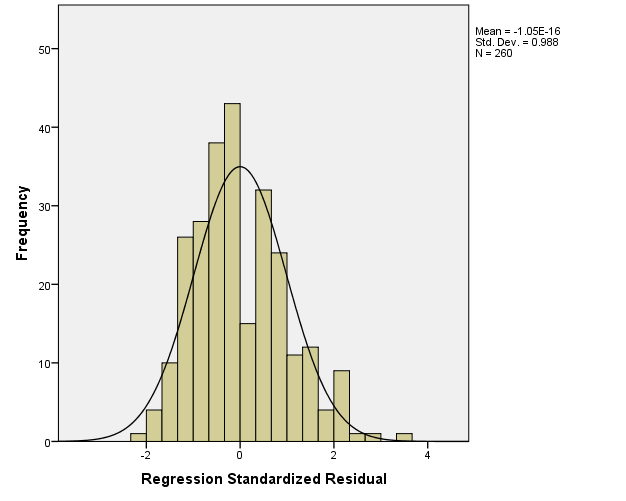


Additional file 1: Figure S3. Histogram of regression standardized residual of the final model

Additional file 1: Table S2. Residuals statistics for the final model

|  | **Minimum** | **Maximum** | **Mean** | **Std. Deviation** | **N** |
| --- | --- | --- | --- | --- | --- |
| **Predicted Value** | 1.65 | 10.53 | 4.98 | 2.076 | 260 |
| **Residual** | -7.572 | 12.820 | .000 | 3.568 | 260 |
| **Std. Predicted Value** | -1.605 | 2.670 | .000 | 1.000 | 260 |
| **Std. Residual** | -2.097 | 3.551 | .000 | .988 | 260 |
